# Engaging Older People in Age-Friendly Cities through Participatory Video Design

**DOI:** 10.3390/ijerph17238977

**Published:** 2020-12-02

**Authors:** Margaret von Faber, Zsuzsu Tavy, Suzan van der Pas

**Affiliations:** 1 Faculty of Social Work & Applied Psychology, University of Applied Sciences Leiden, Zernikedreef 11, 2333 CK Leiden, The Netherlands; Faber.von.m@hsleiden.nl; 2 Chair of Urban Ageing, Faculty of Social Work & Education, The Hague University of Applied Sciences, Johanna Westerdijkplein 75, 2521 EN Den Haag, The Netherlands; Z.K.C.T.Tavy@hhs.nl; 3 Faculty of Health, Nutrition & Sport, The Hague University of Applied Sciences, Johanna Westerdijkplein 75, 2521 EN Den Haag, The Netherlands; 4 Department of Public Health and Primary Care, Leiden University Medical Centre, Hippocratespad 21, 2333 ZD Leiden, The Netherlands

**Keywords:** participatory video design, participation, age-friendly cities

## Abstract

Participatory video design is a novel approach to collect experiences and perceptions of older people about the age-friendliness of their city or neighborhood. In this article, we describe how this co-creative method can add to specific knowledge about the preferences and needs of older people about the improvement or preservation of their environment. We describe two examples of this approach in the cities of The Hague and Leiden, the Netherlands. Persons of 60 years and older were invited to participate in a “workshop” on filmmaking focusing on age-friendly cities. A professional filmmaker and a researcher of the University of Applied Sciences worked in co-creation with older people, to produce short films on the topics that were perceived as important from the perspective of the participants. The older people worked in couples to produce their short films about the city or their neighborhood. Topics of the films included communication and information, outdoor spaces, social relations, and community support. The use of participatory video design can foster empowerment and social interaction among older participants, and insight into the preferences and needs of older people regarding age-friendly cities.

## 1. Introduction

There are two global trends with a major impact on societies in the 21st century: Population ageing and urbanization. World-wide, the proportion of older residents in cities is predicted to increase. Building on the “active ageing” framework, the World Health Organization (WHO) launched the Age-Friendly cities and Communities (AFC) initiative. Eight aspects of city life influencing age-friendliness are specified in a guide: Outdoor spaces and buildings; transportation; housing; social participation; respect and social inclusion; civic participation and employment; communication and information; community support and health services. For a city’s self-assessment, a companion “Checklist of Essential Features of Age-Friendly Cities” can be used to chart progress [1]. An age-friendly city should ideally be a place where older people are actively involved, valued, and supported with infrastructure and services that effectively accommodate their needs [2].

In the Netherlands, the percentage of people of 65 years and over is predicted to increase from the current 19.5% of the Dutch population to 23% in 2030, rising to 25.5% in 2040 [3]. The national policy is to enable older people to live as long as possible in their own home and environment [4]. In the Pact on care for older persons [*Pact voor de Ouderenzorg*] launched in 2018 [5], living environment and housing are important topics. Actions are directed at creating age-friendly living environments. On a local level, municipalities have to record the supply and demand on housing and cooperate with housing corporations, private partners, and citizens. Municipalities also have to gather knowledge on the preferences and needs of older citizens, in order to provide for sufficient housing, alternatives if older people want to move, and the livability of neighborhoods for an increasing older population [4]. In the next decades, the largest increase in older people will be in the major cities and the Western part of the Netherlands [6].

The EU and the local and national governments are emphasizing the need for local communities to encourage older people to play an active role in communities and position them at policy levels as contributors in policies dealing with the local and individual needs that citizens encounter. More tailored solutions centered on older adults’ needs and circumstances need to be developed in collaboration with older adults. In 2014, The Hague was the first city in the Netherlands to join the World Health Organization’s network of age-friendly cities. Together with local stakeholders and various organizations, the city encourages active ageing in the urban environment by stimulating opportunities for activities and social participation in order to enhance quality of life as people age.

In this study, we focus on participation in research, enabling older people to seek their own solutions according to their priorities. The most important difference between participatory research and conventional methodologies is who defines the research problem, and who generates, analyses, owns, and acts on the information that is sought [7]. Participatory research (PR) provides a means to enable older adults to gain ownership over their social and physical environment [8]. PR is used as a term encompassing research approaches such as community-based participatory research, action research (AR), and participatory action research (PAR), which are aimed at engaging community members as co-researchers to guide personal and social change [9].

In participatory research, the target group is actively involved in the research [10]. Different degrees of participation are possible, such as: Identification of themes and relevant research questions, advice on methods and approach of participants, involvement in data gathering, analysis of the data, and dissemination of results. Participatory research as a method is used in several disciplines and academic studies, for example, anthropology, sociology, and psychiatry, but also in the field of health research, education research, and development studies [11]. Moreover, participatory research covers a range of different methodologies in which the role of participants and researcher can be very different. Tangvald-Pedersen and Bongaardt [12] distinguish three different ideologies that have different consequences for user involvement and participation and preferred methodologies. They describe a liberal, market-based ideology; an emancipation-based ideology; and an education-based ideology. In the liberal ideology, the consumer/client is involved as an advisor and consultant. The research design is consultative, the researcher is “in the lead”, and the methodology is important. In the emancipation-based ideology, the research is user-led. The methodology is less prescribed, depending on the goal of the research. The goal of the education-based ideology is to explore and understand the perception of the people under study, for example, a patient or client. The involvement of the participants depends on the context and goal of the research. The research design is collaborative and flexible, adjusting methods to the abilities of the participants. This means that participatory research can be considered a continuum of participation, from projects being led by a professional researcher at one end, to a user-led project at the other end [13]. To evaluate the intended results of the specific research approach, it is essential to describe the knowledge that is collected, to be transparent about the ownership and the distribution of the knowledge.

Participatory research with older people has been conducted for several years. Tanner [14] found that it is even possible to involve older people with dementia in a meaningful way in research processes. Older people with dementia participated in planning the research methods, conducting interviews, and making sense of the findings, with the assistance of the researcher. Both co-researchers and participants can benefit significantly from their participation, but they also make a valuable contribution to the research because they present an “insiders” or “emic” perspective, as opposed to an “outsiders” or “etic” perspective of the matter. Baur and Abma [15] found that active participation of a group of older women in a residential home enhanced empowerment and even co-management of their own needs and preferences. According to the authors, participation can foster empowerment and vice versa, whereby power is not given or taken but is a mutual process that emerges through interaction with others on the level of individuals, organizations, and communities [16]. Buffel [17] described that working with co-researchers on the topic of age-friendly communities also poses challenges, for example, managing expectations about the benefits of the research, or ethical issues of confidentiality between co-researcher and interviewee.

Visual methods, like Photovoice and video research, are specific ways of data-gathering in participatory research. Using the method of Photovoice, older people have participated in research on the age-friendliness in their community [18,19] or the sense of home of nursing home residents [20]. This visual method can be combined with interviews to elicit the views of older people who have more difficulty expressing themselves verbally. Moreover, the use of visual imagery to feature a problem can be particularly empowering and effective in communication with policymakers and stakeholders [18].

In participatory video, participants are trained to use video as part of the research. This practice can be linked to the field of visual anthropology, in which collaborative ethnographic filmmaking has a long history in the work of visual anthropologists David MacDougall and Jean Rouch [21]. This visual method also evolved in social action research, and is used, for example, in development projects and programs [22]. Different forms of, and approaches to, participatory video are possible, varying from organizing a video workshop in a local community and collecting data regarding a certain topic, to finding ways for participants to tell, produce, share, and own their own stories using film. Most approaches have in common that they strive to use film to create a means for often more marginalized or underrepresented groups of people to represent their own experiences [22].

Film is perceived as a powerful tool for representing experiences and communication of knowledge in an experiential way [23,24]. Film also has the ability to capture situations in a social and material context [23,24]. In addition to the power of film as a medium, the process of filmmaking itself is often seen as an important part of the method, and it is held to be able to transform and empower the participants [22]. For participants, this process entails among others, the development of skills, interaction and bonding with others, and reflection on certain topics or on their own identity or position in the community or society at large [22].

The main aim of this paper is to describe how participatory video design can add knowledge about the preferences and needs of older people about the improvement and preservation of their local environment. We describe examples of this approach focusing on age-friendliness in two cities in the Netherlands, The Hague and Leiden. We describe how participants are recruited, the topics that participants perceive as important, and their experiences with this method. In the discussion, we reflect on the implications of this method from a participatory perspective.

## 2. Methodology

Both in The Hague and Leiden, the participatory video design was developed and shaped by a project team, including researchers and filmmakers. In the city of Leiden, representatives of older people were also part of the project team. They decided in which neighborhood the participants would be recruited and assisted in the project. Residents of 60 years or older were eligible for participation, in accordance with the definition of “older persons” of the WHO [1]. As we aimed at a variety of older persons, there were no exclusion criteria on living situation or ethnic background.

### 2.1. The Hague: Setting, Participants, and Method

As part of the activities of The Hague Age-Friendly City, a workshop on documentary making by older people was organized in 2017 [25]. In total, 21 older people (14 women and 7 men), ranging in age from 63 to 90, took part in these workshops. Participants had a Dutch background. Two persons with a Chinese Surinamese background participated.

Over the period of one week, they went through all the phases of making a documentary, forming duos and together making a documentary on “my age-friendly city”. While the topic of “age-friendly city” was chosen by the city council, the participants were free to choose a theme relating to this topic that they further wanted to explore during the workshop. Participants were recruited through an advertisement in *De Posthoorn*, a free local newspaper and via word-of-mouth.

The workshop was conducted over five consecutive days with an introduction starting on Monday and a public premiere at the end of the week on Friday. On the first day, after the introduction, pairs were formed and homework was given for the next day: Decide on a topic and develop a script. On the second day, a test run was done in the studio, with an iPad in hand, to which a hand microphone was connected. On the third day, filming took place at various locations in The Hague, such as a community center, a Chinese co-housing community, and a local gym. The fourth day was devoted to editing the material into short documentaries. Participants were trained in working with Adobe Premiere Pro, and worked on computers in the local movie theater to edit their film.

The workshop was guided by researchers from the University of Applied Sciences Leiden (SP) and The Hague University of Applied Sciences and film professionals with extensive teaching experience, and the latest equipment was used. During the week, they supported the older people in the process of making their films. In the development of the scripts, they gave participants feedback and helped in practicing using the iPads. During the shooting of the film, a film professional was linked to two duos and was there to help and give feedback on the filming and interviewing and to provide technical support if needed. The use of the iPad as a camera posed only a few problems for the older participants. However, editing was more difficult and required more time and work to achieve a coherent end product. During this process, film professionals were there to help. After participants edited their film, the final editing was done by a film professional to create the right sound and volume. On the last day, a premiere took place of the documentaries, where friends and family were invited.

Apart from the individual documentaries that were made, a summary documentary was also made of the entire process of the workshop. All the videos were presented to the municipality, aimed at contributing to aligning policy with the actual needs of older persons when it comes to giving meaning to a (age-friendly) living environment.

### 2.2. Leiden: Setting, Participants, and Method

The University of Applied Sciences Leiden conducts research on the topic of “Living longer in the own environment”. For this topic, researchers (MvF, SP) worked together with the Regional Advisory Board of Older Persons [Ouderenberaad Zuid Holland-Noord] in the city of Leiden. The Regional Advisory Board of Older Persons is a client panel of 15 older persons (aged 60–90 years) who reflect on research proposals/results, housing, and healthcare policy in the Leiden area [26]. In 2017, a participative research on “Vitality and living independently” in the region was conducted. The members of the Regional Advisory Board of Older People were trained as co-researchers and conducted interviews and focus group discussions. However, the report that was presented to the different municipalities with suggestions for improvement did not have the desired impact. In co-creation, the participatory video design was then chosen as a means to focus the attention of policymakers and stakeholders on the perspectives of older people in a specific neighborhood. Members of the board chose the neighborhood for the video project, because it was known that local residents found it important to live and grow older in this place.

Participants were invited to participate by face-to-face contact over a period of three weeks. At a central point in the neighborhood, the local supermarket, members of the Regional Advisory Board of Older Persons, and a researcher (MvF) asked older people to participate in a workshop on filmmaking on the topic of an age-friendly neighborhood. Flyers with an explanation of the goal and content of the workshop were handed out and questions were answered on the spot. In 2019, a group of nine older persons (seven local residents and two members of the Regional Advisory Board of Older People) participated in the project. In total, three women and six men with a Dutch background took part in the workshops. Two other members of the Regional Advisory Board of Older People were present at the meetings.

The workshop was conducted in five half-days. During the first meeting of the workshop, participants received information about the context of the video project, and they explored the topic “Living longer in the own neighborhood”. Subtopics were discussed and participants formed duos on the subjects. In the second meeting, attention was directed at interviewing and filming. Participants rehearsed in interviewing and filming each other, with an iPad in hand, to which a hand microphone was connected. After this, participants were instructed in the use of the app Pinnacle Studio Pro and editing. They practiced in editing their first short films. In the third meeting, participants set off with the iPad and microphone, and filmed in their neighborhood. The professional filmmaker was present for support. The films were edited by the participants, with the help of the filmmaker and his assistant, in the 4th meeting. Finally, in the last meeting, the films were shown to the other participants and the workshop was evaluated. Due to Covid-19 and the following lockdown, it was not possible to organize a public gathering for the presentation of the films in Leiden. However, because some participants expressed the desire to receive their own film, we distributed these to those participants.

## 3. Results

The workshop in The Hague on “My age-friendly city” resulted in 11 mini-documentaries. The workshop in Leiden on “Living longer in the own neighborhood” resulted in 5 mini-documentaries. In the results, we can distinguish the topics that were filmed, and the process of filmmaking.

### 3.1. Topics of the Films

The topics that were filmed were: Outdoor spaces; housing; social participation; communication and information; and community support. Both in The Hague and Leiden, a film with characteristics of the neighborhood was made.

#### 3.1.1. Outdoor Spaces

**Figure 1:**
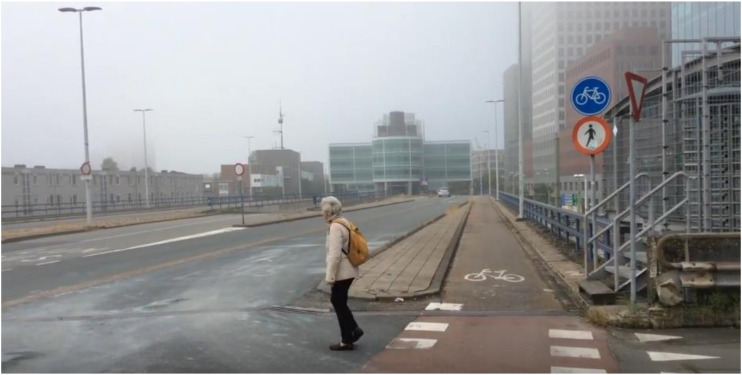
Video fragment: Outdoor spaces in The Hague.

In The Hague, one duo showed the accessibility of the city for older people who travel by foot (see Figure 1). They took the viewer on a trip from point A to point B while evaluating the route from their own perspective. In the video, it is shown that in this short route, big and busy roads and viaducts have to be crossed, sometimes without traffic lights or pedestrian crossings. As one participant tells the viewers: “*You have to be on guard all the time*”.

Another video explored the accessibility of the renovated promenade in Scheveningen beach for people in wheelchairs. The video showed the difficulties a participant in a wheelchair encounters while getting to the promenade from her house. She had to ride over cobblestones, which made her back hurt, and sometimes she would get stuck. The pedestrian bridge was no option for her, as it has two steps at the end. The promenade itself has uneven stone paving, which makes driving in a wheelchair difficult. Another duo made a video on older people visiting a shopping mall in their mobility scooter. They wanted to research the driving skills of older people and found contrasting perspectives. Interviewees driving a mobility scooter mentioned it was easy for them to drive and shop in the shopping mall. By contrast, a shop employee talked about older people lacking driving skills, driving too fast, and crashing into the revolving door. In another video, safety in traffic situations was explored by filming different pedestrian crossings or streets the participants themselves found unsafe. Busy intersections and small streets in which trams and bicycles intersect were shown. The video showed little space between the sidewalk and the tram rails, leaving almost no space for bicycles. One interviewee on a bicycle pointed out that people often get stuck in these tram rails.

Another duo presented a park in the city center with the title “A green oasis of peace”. One interviewee talks about finding peace while walking the small labyrinth in the park. According to the duo and the older people they interviewed in the park, the park provides a pleasant and green space.

In Leiden, the participants that filmed the housing and outdoor spaces reflected on the desire to move to an apartment on the ground level in the same neighborhood. They interviewed a man in a mobility scooter on his view on accessibility. They also filmed bicycles on the pavement and other hindrances, like tree roots, and walking with a walker. With their film, the older participants showed possibilities for improvement in an age-friendly environment.

In another video, participants interviewed a visually impaired woman that also suffered from other physical disabilities. This interviewee experienced difficulties in maintaining her garden. As a solution to this problem, the participants interviewed a young woman who recently started her own small enterprise in helping others with gardening. With the film, they wanted to show this recent opportunity for support in their neighborhood.

#### 3.1.2. Social Participation

In The Hague, one duo portrayed in their video a theater- and dance group for 55+ amateur actors. They showed parts of the rehearsal and performance and interviewed a member of the group. This person explained why she enjoys being part of this group. Another duo investigated what is needed to attract more older people to a community center in the neighborhood, by interviewing people on the street. It becomes clear that people wish to see another ambiance in the center, they want to have good coffee, and they want to participate in other types of activities, like political activities, music, or lectures. In another video, two Chinese participants presented cultural aspects of activities in old age. Filmed in their own house, the viewer sees how they make a religious offering, and also take time to work on calligraphy, which they explain is also a finger exercise. One participant mentioned she was pleased that in The Hague, the Chinese Holidays are celebrated and that she is able to buy Chinese products.

#### 3.1.3. Social Contacts and Community Support

**Figure 2:**
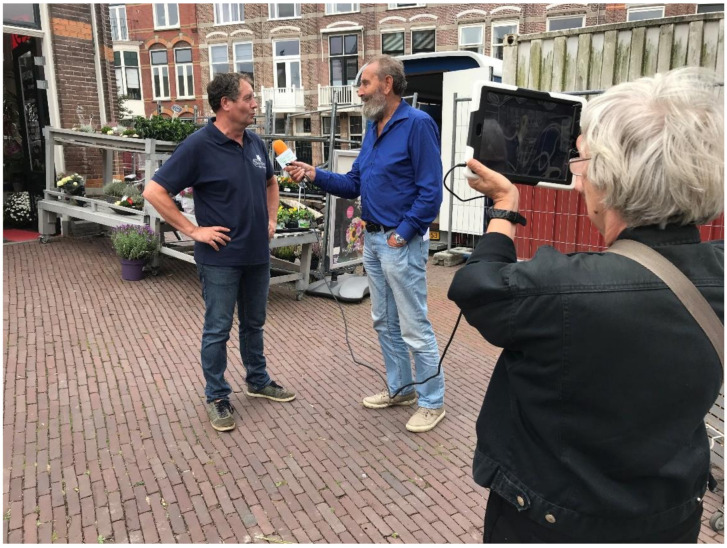
Interviewing a shop-owner in Leiden.

In Leiden, a duo went to the local shops, as shown in Figure 2. They also went to a supermarket and a local restaurant, and discussed social support for older people when they needed assistance in the case of health problems. To their surprise, all interviewees emphasized that they knew their regular customers, that they enquired after their wellbeing when they did not see them for a while, and that they were able to provide services at home (for example, the hairdresser) or deliver food or meals (for example, the catering company and greengrocer) at home.

#### 3.1.4. Communication and Information

In Leiden, one duo chose the theme of information. One participant was actually searching for information on meals and a course in working with a computer. The interviews on the street led to the community center, which could indeed provide an answer to these questions.

#### 3.1.5. Housing, Residential Care, and Social Contacts

In The Hague, a participant filmed a man of 90 years old, one of the participants, on his orientation journey to live in a housing facility for older people. Still living by himself, he is followed to the gym, and he explains what he looks for in a new home: A nice green environment together with the dynamics of the city center, a sports club, a spacious apartment, a recreation room, and nice (older) ladies for social contact.

#### 3.1.6. Characteristics of the Neighborhood

In The Hague, one video showed a relatively new district (Leidschenveen). Participants interviewed passers-by on the street. Although there are activities organized for older people, loneliness and isolation were perceived as important topics in this district. An older lady being interviewed explained how she has to walk 20 min to reach the nearest public transport. It is also shown that to get to the tram at the square of the station, many stairs must be climbed. The participants wanted to show that accessibility of public transport could be improved for older people and persons physically handicapped.

In Leiden, one film described the characteristics of the neighborhood, like the demographics and different types of housing. The positive image of the neighborhood prevailed in the film.

Both in The Hague and Leiden, participants filmed topics perceived as important for an age-friendly city. These topics were often interrelated. The topics of public and outdoor spaces were often linked to mobility and safety. Social participation is also connected to activities that may not be exclusively age-specific, like enjoying a good coffee or listening to music. A connection between social support, keeping an eye on older persons, and the presence of shops and small enterprises in the neighborhood are also valued positively by participants. The film in Leiden shows that a community center can fulfil an important role in providing specific information and practical support for those who are less digitally skilled.

### 3.2. The Process of Participation

In the participation of older persons in the workshops, we can distinguish different aspects: First, participation in the identification of themes and showing what is relevant and important from the perspective of participants; secondly, how the video method itself impacted the empowerment of the participants; thirdly, in making suggestions for improvement; and finally, the follow-up of the films on “owning” the story.

#### 3.2.1. During the Project—Telling the Story

Participants in The Hague had to choose one of the age-friendly city features to explore in their film, whereas in Leiden, the participants were free to choose their own sub-themes. The topics that were picked in Leiden by participants themselves, however, did match the age-friendly city features. They showed what they thought was important. One participant in Leiden stated:

“*I think this topic [community support] is important. I have experienced that myself. I am alone and I go twice a week to the restaurant. They missed me when I was ill. They enquired after my condition. When I returned, I got coffee for free! It is a kind of social concern*”

(woman, Leiden)

In the process of the editing of films, participants could further shape the story they wanted to tell. Participants in The Hague expressed that the support from professional filmmakers during the process was of great value, for it helped them express their views in the best way possible, as some of the participants did find the technical aspects challenging.

**Figure 3:**
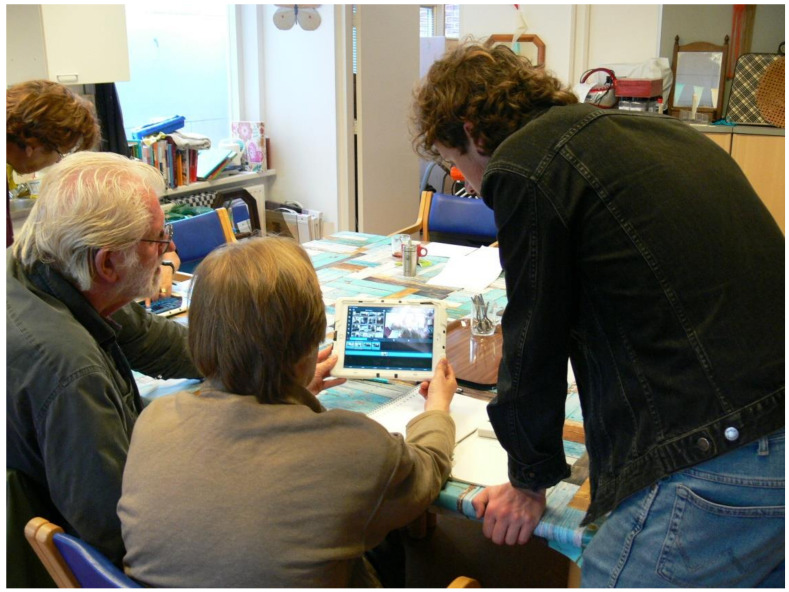
Editing the video fragments in Leiden.

Participants in Leiden were more interested in learning how to film and less in the editing process. They wished to have a say in the final product, but the editing itself was perceived as a bit difficult, despite the received support from the filmmaker and his assistant, as shown in Figure 3. In The Hague, the editing was perceived as challenging too, though one of the participants stated afterward that they did not think they would want to hand over the technical aspects of the editing process though, “*for following every step yourself, makes the whole process more rewarding*”.

The participants in the workshop also involved other older people in the neighborhood in telling the story as well. Participants interviewed people on the streets, or people they already knew on their topic. In one of the videos in The Hague, we followed an acquaintance of one of the participants as she showed her experiences while driving a mobility scooter in the mall. Local organizations in the community were also involved in the process of exploring a topic. In The Hague, for example, we see a theater group for older people, a Chinese co-housing community, a gym, and a community center.

#### 3.2.2. Filmmaking as a Process

Both the participants in The Hague and Leiden expressed enjoying learning something new together, although there were some participants who had previous experience with making a film. Some participants were happy to see that they could learn to use, film, and edit with iPads, something not all of them were expecting when they started the project as one participant who called herself “digitally illiterate” expressed. One participant in The Hague even emphasized the importance of showing the world that “*older people can learn new things too, even new media*.”

**Figure 4:**
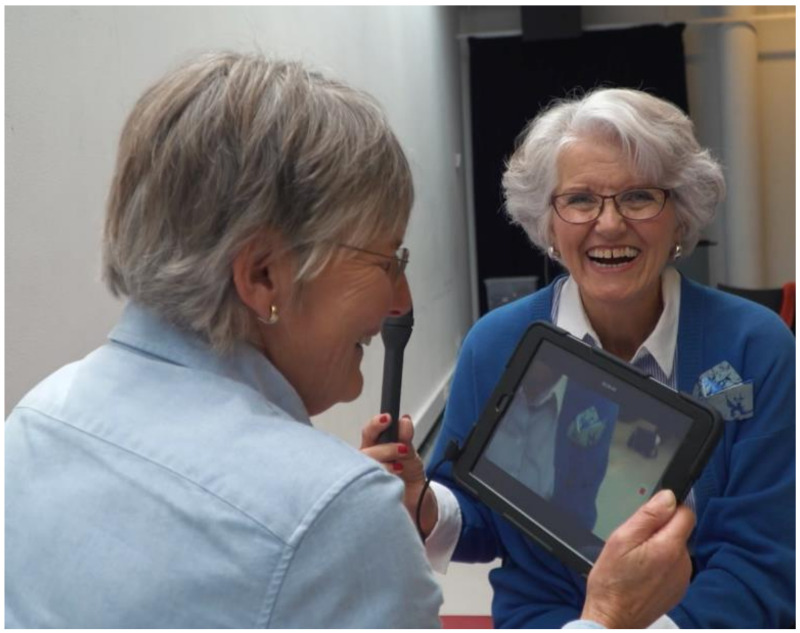
Participants in The Hague having fun together.

During the process, some participants in The Hague formed new friendships. Learning something fun and new together, and experiencing this process during the week together might have attributed to this. Figure 4 shows two participants having fun in rehearsing recording and interviewing. In one of the films, the viewers can see a duo laugh and making jokes together, during a search for housing for older people. This fun is captured and visible for the viewers. Even after the project was finished, this participant and his new friend continued the journey and visited housing for older people together.

Also in Leiden, participants enjoyed working in duos, because they could help and complement each other. They even started filming and interviewing together even outside the scheduled film day, which was possible as they could lend the iPads. One participant was eager to continue in filmmaking, and he kept contact with some of his fellow participants and informed the professional filmmaker afterward. In both cities, several participants kept contact with each other after the workshop finished.

Participants in The Hague expressed being proud of the films they had made, and some of them also expressed feeling good about exploring social themes together. One participant even linked her participation in this project to a newfound drive for societal involvement:

“*I started to feel young again, to feel involved again, and I started to feel I wanted to be part of society again. I experienced different sides of myself again. Looking back, it was the start of a phase in which I started to follow a new direction.*”

(woman, The Hague).

#### 3.2.3. Suggestions for Improvement

In Leiden, the films in themselves created awareness among the participants of what could be improved:

“*I thought the film about the information was the best. “Where do I have to go if I have these questions?”. I think the community center is the place, and maybe we could go one step further and make it a center of knowledge […] there has to be information desk from the municipality. It doesn’t have to be put on a higher level, but on an extended level*.”

(woman, Leiden)

“*In another neighborhood, they have this fitness for older persons. That could also be initiated here*.”

(man, Leiden)

The suggestions for improvement were not only directed at stakeholders in the city, but also to older people themselves:

“*Shop owners stated that they only provided support for regular customers. [… as an older person] you must continue to do your shopping in your own neighborhood and visit the hairdresser, and that kind of things. When you need help, you can call upon them and they will come to you. So this is an advice for older people.*”

(woman, Leiden).

In the evaluation of the process, participants agreed that it was important to make your voice heard as an older person.

#### 3.2.4. After the Filming: Owning the Story

In The Hague, on the last day of the project, a festive premiere took place in the city’s movie theater. Here, the films were proudly shown to friends, family, and involved parties. After the premiere, a group discussion was held about the themes in the films. The themes were further explored and the discussion provided new insights. This process and these insights, however, were not well-documented or included as results. After the project, the films from the project in The Hague were shown on a website and the films were handed over to the municipality by the project team.

After the films were finished, participants in Leiden had little interest in a follow-up by participating in a focus group about the final results and suggestions for improvement. The main aim of the participants was to increase their knowledge on filmmaking. Only two of the older participants were willing to participate in the focus group, which constituted of a member of the Regional Advisory Board of Older People, the chairman of the Neighborhood association, and a representative of the municipality that is responsible for the livability in the neighborhood [the “*wijkregisseur*”]. Suggestions for improvement were discussed on the topics: Information and communication, housing, community health and dementia, mobility, and safety. An example of the latter is to make pavements free of bicycles and other obstructions, and make it wide enough for wheelchairs and walkers.

Participants also made suggestions for relevant stakeholders that could be involved in these improvements. The Neighborhood association could discuss the topic of bicycles on the pavement in the neighborhood-newsletter, and Council officials of the neighborhood and the department of Communication could emphasize the topic of mobility and safety for the city of Leiden. The municipality could also improve the pavements. The Community center could take up a central role as provider of information, together with the Neighborhood-newsletter. Health providers could provide an update of the available printed information on services for older people and informal caregivers. Municipality and social housing associations are important for the improvements in the topics concerning housing and modification. Healthcare providers, welfare organizations, and case managers could provide information on neighborhood level, for example, on dementia and support for informal caregivers. Healthcare providers could offer fitness, Tai-Chi, or other courses in the neighborhood. The primary school in the neighborhood could be involved by offering space for these courses.

Covid-19 and the following lockdown prevented the presentation of the films. Moreover, changes in stakeholders, due to public procurement for welfare organizations, provided an impediment for the discussion on the suggestions for improvement. However, the Neighborhood association and the “*wijkregisseur*” have already taken up the responsibility of improving the mobility and safety on pavements, and the improvement for information and communication.

## 4. Discussion

By applying the method of participatory video design, the aim of this study was to investigate the preferences and needs of older people about the age-friendliness of their environment. This method enabled older people to show aspects of age-friendliness from their own perspective. The participants chose the following topics: Outdoor spaces, housing, social participation, communication and information, and community support. These topics are in accordance with elements of the model of an age-friendly city of the WHO [1]. The films showed the relation between participants and their environment. Not only did the films show what could be improved, but also what was highly appreciated and what should be preserved as an aspect of age-friendliness, like the support of local shop owners. In relation to improvement of age-friendliness, the activities in the community center were mentioned, as well as communication and information. Most public information is made available in a digital form, and the digital competence of older people is stimulated. However, one of the videos showed that oral and printed information remain essential for older persons that are not (or no longer) digitally skilled. With regard to housing, mobility, and safety, participants showed environmental barriers and risks, such as busy roads without traffic lights or pedestrian crossings, or sidewalk hazards with little space that poses a problem for a person with a walker. As we follow one of the participants in The Hague on her trip from point A to B, we can see she encounters different obstacles. The viewer gets a sense of the whole route, the imposing environment, and the reaction of the participants while being confronted with this environment (Figure 1). This is perceived as one of the strengths of film.

Apart from the insight into the various aspects of age-friendliness, the method of participatory video added to the collection of data that were unexpected and creative. According to MacDougall [23], exploring the neighborhood through the lens of a camera can provide participants with a new view, as one engages with the world in other ways. In this research, participants were conscious or became more aware of the age-friendliness of their environment. For example, in the focus group in Leiden, participants stated that they were unaware of the support for older people living with dementia in their neighborhood.

The process of filmmaking was an important aspect for participants. Moreover, statements on “*feeling part of society*” and “*enabling the voice of older people to be heard*” resembled elements of empowerment. Our research design can be described as consultative, stemming from a liberal ideology. Our stakeholders depart from the standpoint that knowing the preferences of the older citizens leads to better improvements in age-friendliness of the city. However, the results of this research show that they also could tie in with an emancipatory ideology [12].

A potential limitation of visual methods like video research is that it could exclude persons with impairments related to vision, hearing, or mobility. This could introduce a selection bias and under-representation of vulnerable groups [19]. These groups are likely to include a disproportionate share of people from lower socio-economic backgrounds, and people with mental health issues, low self-esteem, and health problems [27]. However, because in visual methods, like video design and photovoice, language is not the only element, it has been documented that it can also be used with populations in different health and living conditions, for example, people with dementia [20], different social economic status, or immigrants [27]. This participatory video design attracted older people that were interested in learning something new, and although participants were sometimes insecure about their ability to make a documentary, they also perceived it as a challenge. Both in The Hague and Leiden, some participants had filmed in their youth, and were eager to learn how to film with an iPad and edit the product. For those who found the editing the most difficult part, professional support was at hand. As mentioned in other participatory research, the training contributed to older participants’ “skills and self-esteem and a sense of mastery” [18,22,28]. We found that participants were very proud of their achievements. Working in duos also resulted in mutual learning. This aspect can contribute to a “stronger voice” of older people in processes of co-creation for improvement [15].

The films showed the heterogeneity in older people, challenging the dominant stereotypes of older people as frail and dependent. For future research, it would be interesting to investigate the effects of these images from the perspective of the viewers. For example, exploring the effects on policy makers could reveal to what extent these images would influence policy advice.

Video research is able to change the traditional role of researcher and the “researched” [29]. The participants may act as “reflective insider” and as “active researcher”, interviewing other people. New roles arise, for example, the “*director*” and the “*editor*” [30]. Both in The Hague and Leiden, participants took up a role as researcher, eliciting the aspects that they wanted others to see. In the process of editing, some participants in Leiden were leaving out “unwanted” comments, because of strategic reasons. These participants thought the local community center was of major importance and some of the participants were actively involved in activities of this community center. However, in one interview, the interviewee stated that the community center was for “the most vulnerable persons” in the neighborhood and he distanced himself from this negative image. This, as a negative perceived remark, was deliberately left out in the montage by the participants. These findings are relevant because what is deliberately left out of the film might have been used for improvement, and at the same time, it underscores the fear of the participants of depicting negative images of aspects that they find important to keep within the neighborhood.

A challenge for video research is how to “use” the data for improvement of the neighborhood and city. In order to have a maximum impact, this kind of research needs to be embedded in AFC initiatives from the start, with the municipality as a committed and proactive partner. Buffel [17] describes how working with older co-researchers on age-friendly communities in Manchester (UK) was embedded in a partnership between the Manchester City Council, the University of Manchester, various community organizations, and older people, committed to the goal of developing “age-friendly communities.” The aspiration for the dissemination of results and acting upon suggestions has to be a product of co-creation between researchers and stakeholders, like a city council. Co-creation can be seen as a practice of interactions between older adults, public professionals, students, researchers, and community stakeholders who jointly define needs and choices, as well as design and implement services and support [31]. Co-creation is when people relate to each other and interact in defining, designing, and implementing a particular service, product, or practice [32]. In the literature, a distinction can be made between three types of involvement: (1) Citizens as co-implementer of public policy, (2) citizens as co-designer, and (3) citizens as co-initiator [32]. The first level is represented most frequently, which involves the citizen as co-implementer of the public service. Here, citizen involvement has already been defined by the public service. The second level approaches the citizen as co-designer of how the product or service should be delivered. In most cases, the initiative for the co-creation lies with the public institution, but citizens decide how the service is being delivered. The third level represents the citizen as initiator and the government as supporting actor. In our video projects, the role of the municipalities was different.

In The Hague, the municipality was setting up a platform “Age-Friendly City.” The municipality launched a multitude of new projects, but it was unclear how the results could be embedded. No arrangements were made to evaluate and discuss the results of the video research. In the final phase of the research, a dialogue between participants, stakeholders, and the municipality would have benefited the follow up [19]. This would have provided an opportunity for improvements and could have fostered empowerment of older people.

In Leiden, not the City council, but the Regional Advisory Board of Older People was co-designer of the research and participated in the research process. Their main aim was to communicate the preferences and needs of older people to policy-makers and stakeholders, like social housing organizations. A short documentary was perceived as more effective than written reports [18]. However, due to Covid-19, we are still in the process of discussing the results of the video research with the municipality, the neighborhood association, and stakeholders. At the neighborhood level, some improvements have already been made. As the neighborhood association is publishing the video research and the improvements for all neighborhood residents in the local newspaper, this may enhance social interaction and feelings of empowerment of the participants. Recently, the municipality has launched a plan to increase age-friendliness of the city, so this provides opportunities to discuss the results and suggestions for improvements on a local level. When we compare the two different approaches, we can conclude that the effect of video research in the context of age-friendly cities is related to the way it is embedded in local policies and agreements with stakeholders on a local level.

### Lessons Learned

For future research, it is essential to make some improvements. First, it is important to announce the method in a different way to participants. Instead of focusing on the workshop or video documentaries, participants could be made aware that the results can be used for improvement in an age-friendly city. The focus group could be at the start of the project, discussing the topics more in depth and then focusing on making a documentary on a subtheme. A second focus group could be included to discuss findings and suggestions for stakeholders and the municipality [19]. It is also important to acknowledge that co-creation processes need to address possible barriers both on the organizational level and on the citizen level. On the organizational side, this refers to, for instance, policies that support co-creation, and on the citizen side, this might be the lowering of thresholds for citizens to participate [32]. A prerequisite for co-creation and use of the results is to involve the civil servants from the local municipality and other stakeholders from the start.

Secondly, it is important to discuss with participants the roles and responsibilities, and involve them in the design of the process. For example, instead of editing the films and making a short documentary, more professional assistance in the editing process can be positive for their involvement in the process.

Another important aspect is that participants in both projects in The Hague and Leiden stated that they needed more time for the actual filming. Improvement in a longer period of time, months instead of weeks, may result in more reflection by participants on the theme, the script, and the results, instead of focusing on only learning “something new.” It can also enhance the process of filmmaking for the participants. Moreover, it may be beneficial for the involvement of the municipalities and other stakeholders as partners in co-creation and the follow-up process.

These improvements may benefit future video research with older people. In The Hague, one year later, a follow-up project was organized; a new group of participants started with the topic “meaningful ageing”. In Leiden, there are also plans for future video research on age-friendliness in another neighborhood.

## 5. Conclusions

Participatory video research can provide rich data about the age-friendliness of cities and the experiences, preferences, and needs of older people living in these cities and neighborhoods. Older participants showed their own experiences and involved other older people.

The use of participatory video design gives a voice to older people that are interested in a more active approach to research. It also fosters a positive image of older adults as active and motivated persons to improve their living environment. In video research, older people fulfill different roles in the choice of topics, in telling the story, and in directing and editing the videos for a documentary that may lead to improvements. A prerequisite for the follow-up for improvements in age-friendliness is a process of co-creation with relevant stakeholders from the start.

## Declaration

Participants of the film projects of the University of Applied Sciences Leiden and The Hague University of Applied Sciences have signed the informed consent, and gave us permission to use the photos taken during the project as illustration in a scientific publication about the film project. The article discusses how film can be used to learn more about the experiences of the elderly and how senior-friendly the city is. The photos are for illustration purposes in the article.

## References

[1] World Health Organization (2007). Global Age-Friendly Cities: A Guide.

[2] Van Hoof J., Dikken J., Buttigieg S.C., van den Hoven R., Kroon E., Martson H.R. (2020). Age-friendly cities in the Netherlands: An explorative study of facilitators and hindrances in the built environment and ageism in design. Indoor Built Environ..

[3] CBS Prognose Bevolking; Kerncijfers, 2019–2060. https://opendata.cbs.nl/statline/#/CBS/nl/dataset/84645NED/table?ts=1594391644164.

[4] Rijksoverheid Programma Langer Thuis. https://www.rijksoverheid.nl/documenten/rapporten/2018/06/15/programma-langer-thuis.

[5] Rijksoverheid Pact Voor de Ouderenzorg. https://www.rijksoverheid.nl/documenten/publicaties/2018/03/08/pact-voor-de-ouderenzorg.

[6] Planbureau Voor de Leefomgeving Regionale Bevolkings- en Huishoudensprognose. https://themasites.pbl.nl/o/regionale-bevolkingsprognose/.

[7] Cornwall A., Jewkes R. (1995). What is participatory research?. Soc. Sci Med..

[8] Corrado A.M., Benjamin-Thomas T.E., McGrath C., Hand C., Laliberte Rudman D. (2020). Participatory Action Research with Older Adults: A Critical Interpretive Synthesis. Gerontologist.

[9] Cargo M., Mercer S.L. (2008). The Value and Challenges of Participatory Research: Strengthening Its Practice. Annu. Rev. Public Health.

[10] Smaling A. (2009). Participatief onderzoek: Een overzicht. KWALON.

[11] Lake D., Wendland J. (2018). Practical, Epistemological, and Ethical Challenges of Participatory Action Research: A Cross-Disciplinary Review of the Literature. J. High. Educ. Outreach Engagem..

[12] Tangvald-Pedersen O., Bongaardt R. (2017). Towards a tinkering participatory research method in mental health. Scand. J. Disabil. Res..

[13] Fenge L. (2010). Striving towards Inclusive Research: An Example of Participatory Action Research with Older Lesbians and Gay Men. Brit. J. Soc. Work.

[14] Tanner D. (2012). Co-Research with older people with dementia: Experience and reflections. J. Mental Health.

[15] Baur V., Abma T. (2012). ‘The Taste Buddies’: Participation and empowerment in a residential home for older people. Ageing Soc..

[16] Zimmerman M.A., Rappaport J., Seidman E. (2000). Empowerment theory. Psychological, organizational and community levels of analysis. Handbook of Community Psychology.

[17] Buffel T. (2018). Older Coresearchers Exploring Age-Friendly Communities: An “Insider” Perspective on the Benefits and Challenges of Peer-Research. Gerontologist.

[18] Novek S., Morris-Oswald T., Menec V. (2012). Using photovoice with older adults: Some methodological strengths and issues. Ageing Soc..

[19] Ronzi S., Pope D., Orton L., Bruce N. (2016). Using photovoice methods to explore older people’s perceptions of respect and social inclusion in cities: Opportunities, challenges and solutions. SSM Popul. Health.

[20] Van Hoof J., Verhagen M.M., Wouters E.J.M., Marston H.R., Rijnaard M.D., Janssen B.M. (2015). Picture Your Nursing Home: Exploring the Sense of Home of Older Residents through Photography. J. Aging Res..

[21] Pink S., Pink S. (2007). Visual Interventions: Applied Visual Anthropology.

[22] White S.A., White S.A. (2003). Participatory Video: A Process that Transforms the Self and the Other. Participatory Video: Images that Transform and Empower.

[23] MacDougall D. (2006). The Corporeal Image: Film, Ethnography, and the Senses.

[24] MacDougall D. (1998). Transcultural Cinema.

[25] Van Vliet J., Ligthart M., Boon M., van der Pas S. (2018). Leeftijdsvriendelijke stad in beeld. Documentaires maken met ouderen. Geron.

[26] Van Blijswijk S., de Waard C.S., van Peet P.G., Keizer D., von Faber M., de Waal M., den Elzen W., Gussekloo J., Blom J.W. (2018). Wishes and needs of community-dwelling older persons concerning general practice: A qualitative study. PLoS ONE.

[27] Mysyuk Y., Huisman M. (2019). Photovoice method with older persons: A review. Ageing Soc..

[28] Blair T., Minkler M. (2009). Participatory Action Research with Older Adults: Key Principles in Practice. Gerontologist.

[29] Whiting R., Symon G., Roby H., Chamakiotis P. (2018). Who’s Behind the Lens? A Reflexive Analysis of Roles in Participatory Video Research. Organ. Res. Methods.

[30] Gibson B.E. (2005). Co-Producing Video Diaries: The Presence of the “Absent” Researcher. Int. J. Qual. Methods.

[31] Van der Pas S. (2017). Engaging ageing communities as co-creators of social services and support. Innov. Aging.

[32] Voorberg W.H., Bekkers W.J.J.M., Tummers L.G. (2015). A systematic review of co-creation and co-production: Embarking on the social innovation journey. Public Manag. Rev..

